# Ginkgolic Acid is a Multi-Target Inhibitor of Key Enzymes in Pro-Inflammatory Lipid Mediator Biosynthesis

**DOI:** 10.3389/fphar.2019.00797

**Published:** 2019-07-17

**Authors:** Jana Gerstmeier, Julia Seegers, Finja Witt, Birgit Waltenberger, Veronika Temml, Judith M. Rollinger, Hermann Stuppner, Andreas Koeberle, Daniela Schuster, Oliver Werz

**Affiliations:** ^1^Chair of Pharmaceutical/Medicinal Chemistry, Institute of Pharmacy, Friedrich-Schiller-University Jena, Jena, Germany; ^2^Department of Pharmaceutical Analytics, Pharmaceutical Institute, Eberhard-Karls-University Tuebingen, Tuebingen, Germany; ^3^Institute of Pharmacy/Pharmacognosy and Center for Molecular Biosciences Innsbruck (CMBI), University of Innsbruck, Innsbruck, Austria; ^4^Department of Pharmacognosy, Faculty of Life Sciences, University of Vienna, Vienna, Austria; ^5^Institute of Pharmacy, Department of Pharmaceutical and Medicinal Chemistry, Paracelsus Medical University Salzburg, Salzburg, Austria

**Keywords:** ginkgolic acid, microsomal prostaglandin E2 synthase-1, 5-lipoxygenase, cyclooxygenase, multi-target inhibitor, lipid mediator

## Abstract

**Introduction:** Lipid mediators (LMs) comprise bioactive metabolites of polyunsaturated fatty acids, including pro-inflammatory prostaglandins (PGs), thromboxanes (TXs), and leukotrienes (LTs), as well as specialized pro-resolving mediators (SPMs). They are essentially biosynthesized *via* cyclooxygenase (COX) and lipoxygenase (LO) pathways in complex networks and regulate the progression as well as the resolution of inflammatory disorders including inflammation-triggered cancer. Ginkgolic acid (GA) is a phenolic acid contained in *Ginkgo biloba* L. with neuroprotective, antimicrobial, and antitumoral properties. Although LMs regulate microbial infections and tumor progression, whether GA affects LM biosynthesis is unknown and was investigated here in detail.

**Methods:** Pharmacophore-based virtual screening was performed along with docking simulations. Activity assays were conducted for isolated human recombinant 5-LO, cytosolic phospholipase (PLA)_2_α, COX-2, and ovine COX-1. The activity of human mPGES-1 and thromboxane A_2_ synthase (TXAS) was determined in crude cellular fractions. Cellular LM formation was studied using human monocytes, neutrophils, platelets, and M1- and M2-like macrophages. LMs were identified after (ultra)high-performance liquid chromatography by UV detection or ESI-tandem mass spectrometry.

**Results:** GA was identified as virtual hit in an mPGES-1 pharmacophore-based virtual screening. Cell-free assays revealed potent suppression of mPGES-1 activity (IC_50_ = 0.7 µM) that is fully reversible and essentially independent of the substrate concentration. Moreover, cell-free assays revealed COX-1 and TXAS as additional targets of GA with lower affinity (IC_50_ = 8.1 and 5.2 µM). Notably, 5-LO, the key enzyme in LT biosynthesis, was potently inhibited by GA (IC_50_ = 0.2 µM) in a reversible and substrate-independent manner. Docking simulations support the molecular interaction of GA with mPGES-1 and 5-LO and suggest concrete binding sites. Interestingly, interference of GA with mPGES-1, COX-1, TXAS, and 5-LO was evident also in intact cells with IC_50_ values of 2.1–3.8 µM; no radical scavenging or cytotoxic properties were obvious. Analysis of LM profiles from bacteria-stimulated human M1- and M2-like macrophages confirmed the multi-target features of GA and revealed LM redirection towards the formation of 12-/15-LO products including SPM.

**Conclusions:** We reveal GA as potent multi-target inhibitor of key enzymes in the biosynthesis of pro-inflammatory LMs that contribute to the complex pharmacological and toxicological properties of GA.

## Introduction

Lipid mediators (LMs) comprise polyunsaturated fatty acid (PUFA)-derived metabolites such as pro-inflammatory prostaglandins (PGs), thromboxanes (TXs), and leukotrienes (LTs), as well as specialized pro-resolving mediators (SPMs) that critically regulate the inflammatory response (Serhan, 2014). One hallmark of unresolved inflammation is constantly elevated levels of PGs and LTs, leading to chronic diseases like asthma, cardiovascular diseases, Alzheimer’s disease, type 2 diabetes, and cancer (Tabas and Glass, 2013). These pro-inflammatory LMs act mainly *via* distinct G protein-coupled receptors (GPCRs) on target cells (Im, 2013) and are produced from free arachidonic acid (AA) within organized enzyme cascades (Funk, 2001).

Cyclooxygenases (COX)-1/2 transform AA into PGH_2_ that is further converted to the bioactive prostanoids PGD_2_, PGE_2_, PGF_2α_, PGI_2_, and TXA_2_ by specialized PG or TX synthases, respectively (Dubois et al., 1998). PGE_2_ is involved in inflammation, fever, and pain and also protects the gastrointestinal mucosa and regulates natriuresis, blood pressure, and ovulation. It is produced by three different PGE_2_ synthases (PGES), namely, cytosolic PGES (cPGES), microsomal PGES (mPGES)-1, and mPGES-2 (Khanapure et al., 2007; Koeberle and Werz, 2015). The inducible mPGES-1 is believed to be mainly responsible for massive PGE_2_ biosynthesis at inflammatory sites and is thus considered as an attractive target for intervention with inflammation-related disorders including also cancer (Koeberle and Werz, 2015).

Besides COXs, 5-lipoxygenase (5-LO) also contributes to the formation of pro-inflammatory eicosanoids, i.e., the LTs (Radmark et al., 2015). 5-LO converts AA into the epoxide LTA_4_ that is hydrolyzed by LTA_4_ hydrolase to LTB_4_ or conjugated with glutathione by LTC_4_ synthase to LTC_4_, and further processed to LTD_4_ and LTE_4_ (cys-LTs). While LTB_4_ is a chemoattractant and activates phagocytes, the cys-LTs cause broncho- and vasoconstriction and increase vascular leakage (Funk, 2001). Accordingly, 5-LO and LT have long been implicated in asthma, allergic rhinitis, and arthritis as well as in cardiovascular disease and cancer (Werz, 2002).

In addition to these pro-inflammatory eicosanoids, specialized pro-resolving mediators (SPMs) are biosynthesized from PUFAs such as eicosapentaenoic acid (EPA) and docosahexaenoic acid (DHA) involving COX/LO pathways. The SPM superfamily includes lipoxins (LXs) biosynthesized from AA, E-series resolvins (Rvs) from EPA, and DHA-derived D-series resolvins, protectins, and maresins that actively stop inflammation to promote resolution of inflammation and tissue regeneration (Serhan, 2014).

Based on the pro-inflammatory actions of PGs and LTs, pharmacological concepts pursue suppression of eicosanoid formation during inflammatory disorders. Because AA-converting cascades act in conjunction, blockade of the COX pathway by non-steroidal anti-inflammatory drugs (NSAIDs) suppresses the early inflammatory response caused by PGE_2_ (Rainsford, 2007) but may also promote a shift towards LT biosynthesis that boosts cardiovascular and gastrointestinal side effects or induces bronchoconstriction (Rainsford, 1993; Burnett and Levy, 2012). Novel pharmacological strategies focus on dual or multiple target concepts, such as dual mPGES-1/5-LO inhibitors (Koeberle and Werz, 2015). In fact, mPGES-1 and 5-LO pharmacophore models have been established that in combination with virtual screening approaches have led to the identification of various small molecules that dually inhibit both PGE_2_ and LT synthesis (Koeberle et al., 2016; Koeberle and Werz, 2018).

In search of chemotypes as mPGES-1 inhibitors, we identified ginkgolic acid (GA), a 6-alkenyl derivative of salicylic acid contained in *Ginkgo biloba*, from an in-house Chinese Herbal Medicine (CHM) database comprising 10,216 natural products as active molecules using two established mPGES-1 pharmacophore models and virtual screening approaches (Fakhrudin et al., 2010; Waltenberger et al., 2011; Li et al., 2014). Many beneficial properties have been described for GA such as anti-tumoral (Fukuda et al., 2009; Zhou et al., 2010) and antibacterial (Hua et al., 2017) effects as well as suppression of inflammation along with reduced COX-2 expression and PGE_2_ levels in human umbilical vein endothelial cells (Li et al., 2018). However, direct molecular targets of GA related to inflammation or LM formation are unknown. We show here that GA potently inhibits the activities of multiple enzymes in eicosanoid biosynthesis, besides mPGES-1, in cell-free and cell-based assays, and we suggest that these actions may contribute to the bioactivities of GA shedding light on its molecular pharmacological and toxicological profile.

## Materials and Methods

### Materials

GA (purity ≥ 98%) was a gift from Dr. Willmar Schwabe GmbH & Co. KG (Karslruhe, Germany). Zileuton [*N*-(1-benzo[b]thien-2-ylethyl)-*N*-hydroxyurea] was from Sequoia Research Products (Oxford, UK); PGH_2_ was from Larodan (Malmö, Sweden); IL-1β was from ReproTech (Hamburg, Germany); RSC-3388 was from Calbiochem (Darmstadt, Germany); EDTA and Nonidet P-40 were from AppliChem (Darmstadt, Germany); p-anisidinium chloride was from Merck (Darmstadt, Germany); Insect Express Sf9-S2 and RPMI media, glutamine, penicillin, and streptomycin were from PAA (Coelbe, Germany); Bac-to-Bac baculovirus expression system was from Invitrogen (Karlsruhe, Germany); Ni-NTA agarose was from Qiagen (Hilden, Germany). AA, Ca^2+^-ionophore A23187, dextrane, dithiothreitol, fetal calf serum, indomethacin, lipopolysaccharide (LPS), Triton X-100, and all other chemicals were purchased from Sigma-Aldrich (Taufkirchen, Germany), unless stated otherwise. HPLC/UPLC solvents were from VWR (Darmstadt, Germany).

### Cells and Cell Isolation

Human peripheral blood (University Hospital Jena, Germany) was withdrawn from fasted (12 h) healthy adult donors (18–65 years) that had not taken any anti-inflammatory drugs during the last 10 days, by venipuncture in heparinized tubes (16 IE heparin/ml blood), with written informed consent. The experimental protocol was approved by the ethical committee of the University Hospital Jena. All methods were performed in accordance with the relevant guidelines and regulations. The blood was centrifuged at 4,000 × *g* for 20 min at 20°C for preparation of leukocyte concentrates. Leukocyte concentrates were then subjected to dextran sedimentation and centrifugation on Ficoll-Histopaque 1077-1 (Sigma-Aldrich) cushions. To isolate platelets, the supernatants were mixed with phosphate-buffered saline (PBS) pH 5.9 (3:2 v/v) and centrifuged (2,100 × g, 15 min, 20°C), and the pelleted platelets were resuspended in PBS pH 5.9/0.9% NaCl (1:1, v/v). The washed platelets were finally resuspended in PBS pH 7.4 and 1 mM CaCl_2_. To isolate polymorphonuclear leukocytes (PMNLs), the contaminating erythrocytes of pelleted PMNLs were lysed by hypotonic lysis. PMNLs were then washed twice in ice-cold PBS and finally resuspended in PBS pH 7.4 containing 1 mg/ml glucose and 1 mM CaCl_2_ (PGC buffer). Monocytes were isolated from peripheral blood mononuclear cell fraction that was obtained after Ficoll-Histopaque 1077-1 centrifugation of leukocyte concentrates, by adherence for 1.5 h at 37°C to culture flasks (Greiner, Nuertingen, Germany). The cell density was 2 × 10^7^ cells/ml RPMI 1640 medium containing 2 mM *L*-glutamine and 100 U/ml penicillin and 100 µg/ml streptomycin, and the purity was >85%, defined by forward- and side-light scatter properties and detection of the CD14 surface molecule by flow cytometry (BD FACSCalibur, Heidelberg, Germany). The monocytes were finally resuspended in ice-cold PBS plus 1 mg/ml glucose or in PGC buffer.

For analysis of acute cytotoxicity of GA during pre-incubation periods (i.e., 30 min), the viability of PMNL and monocytes was analyzed by trypan blue exclusion using a Vi-cell counter (Beckmann Coulter GmbH, Krefeld, Germany). Cell viability analysis of A549 cells after 24 and 48 h of exposure to GA was assessed by MTT assay as previously described (Koeberle et al., 2008).

### Differentiation of Monocytes to Macrophages and Macrophage Polarization

The differentiation of monocytes to macrophages and polarization towards M1 and M2 was performed as previously described (Werz et al., 2018). M1 were generated by incubating monocytes with 20 ng/ml GM-CSF (Peprotech, Hamburg, Germany) for 6 days in RPMI 1640 supplemented with 10% FCS, 2 mmol/L *L*-glutamine, penicillin (100 U/ml), and streptomycin (100 µg/ml), followed by 100 ng/ml LPS and 20 ng/ml INF-γ (Peprotech) treatment for another 48 h. M2 were obtained by incubating monocytes with 20 ng/ml M-CSF (Peprotech) for 6 days and subsequent treatment with 20 ng/ml IL-4 (Peprotech) for additional 48 h. Correct polarization and purity of macrophages were routinely checked by flow cytometry (FACS Canto Plus flow cytometer, BD Bioscience) as previously reported (Werner et al., 2019) using the following antibodies: FITC anti-human CD14 (2 µg/test, clone M5E2, BD Bioscience), PE anti-human CD54 (1 µg/test, clone HA58, BD Bioscience), APC-H7 anti-human CD80 (0.25 µg/test, clone L307.4, BD Bioscience), PE-Cy7 anti-human CD163 (2 µg/test, clone RM3/1, Biolegend, San Diego, CA), and PerCP-eFluor710 anti-human CD206 (0.06 µg/test, clone 19.2, Biosciences, San Diego, CA).

### Expression, Purification, and Determination of the Activity of Human Recombinant 5-LO

*E. coli* (BL21) was transformed with pT3-5-LO plasmid, and recombinant 5-LO protein was expressed at 30°C as previously described (Fischer et al., 2003). Cells were lysed in 50 mM triethanolamine/HCl pH 8.0, 5 mM EDTA, soybean trypsin inhibitor (60 µg/ml), 1 mM phenylmethanesulfonyl fluoride, and lysozyme (1 mg/ml), homogenized by sonication (3 × 15 s), and centrifuged at 40,000 × *g* for 20 min at 4°C. The 40,000 × *g* supernatant (S40) was applied to an ATP-agarose column to partially purify 5-LO as described previously (Fischer et al., 2003). Semi-purified 5-LO (0.5 µg) was diluted with ice-cold PBS containing 1 mM EDTA, and 1 mM ATP was added; the final volume was 1 ml. Samples were pre-incubated with the test compounds or vehicle (0.1% DMSO) as indicated. After 10 min at 4°C, samples were pre-warmed for 30 s at 37°C, and 2 mM CaCl_2_ plus the indicated concentration of AA was added to start 5-LO product formation. The reaction was stopped after 10 min at 37°C by addition of 1 ml of ice-cold methanol, and the formed metabolites were analyzed by RP-HPLC as previously described (Fischer et al., 2003). 5-LO products include the all-trans isomers of LTB_4_ as well as 5(*S*)-hydroperoxy-6-*trans*-8,11,14-*cis*-eicosatetraenoic acid (5-HPETE) and its corresponding alcohol 5(*S*)-hydroxy-6-*trans*-8,11,14-*cis*-eicosatetraenoic acid (5-HETE).

### Determination of Lipoxygenase Products in Intact Cells

For determination of LO products in intact PMNL, cells (2 × 10^6^) were resuspended in 1 ml of PGC buffer, preincubated for 15 min at 37°C with test compounds or vehicle (0.1% DMSO), and incubated for 10 min at 37°C with the indicated stimuli. Thus, the Ca^2+^-ionophore A23187 (2.5 µM) was added with or without 20 µM AA, and 10 min later, the reaction was stopped on ice by addition of 1 ml of methanol. Then, 30 µl of 1 N HCL, 500 µl of PBS, and 200 ng of prostaglandin B_1_ were added and the samples were subjected to solid-phase extraction on C18 columns (100 mg, UCT, Bristol, PA, USA). 5-LO products (LTB_4_ and its *trans*-isomers, 5-HETE), the COX-1 product 12(*S*)-hydroxy-5-*cis*-8,10-*trans*-heptadecatrienoic acid (12-HHT), and the 12- and 15-LO products 12(*S*)-hydroxy-6-*trans*-8,11,14-*cis*-eicosatetraenoic acid (12-HETE) and 15(*S*)-hydroxy-5,8,11-*cis*-,13-trans-eicosatetraenoic acid (15-HETE), respectively, were analyzed by HPLC, and quantities were calculated on the basis of the internal standard PGB_1_ as previously described (Steinhilber et al., 1989).

### Preparation of Crude mPGES-1 in Microsomes of A549 Cells and Determination of PGE_2_ Synthase Activity

Preparations of A549 cells and determination of mPGES-1 activity was performed as described previously (Koeberle et al., 2008). In brief, cells were treated with 2 ng/ml IL-1β for 72 h at 37°C, 5% CO_2_. Cells were harvested, sonicated, and homogenized (homogenization buffer: 0.1 M potassium phosphate buffer, pH 7.4, 1 mM phenylmethanesulfonyl fluoride, 60 µg/ml soybean trypsin inhibitor, 1 µg/ml leupeptin, 2.5 mM glutathione, and 250 mM sucrose). The homogenate was subjected to differential centrifugation at 10,000 × *g* for 10 min and 174,000 × *g* for 1 h at 4°C. The pellet (microsomal fraction) was resuspended in 1 ml of homogenization buffer, and the total protein concentration was determined by using DC Protein Assay (Bio-Rad, Munich, Germany). Microsomal membranes were diluted in potassium phosphate buffer (0.1 M, pH 7.4) containing 2.5 mM glutathione. Test compounds or vehicle were added, and after 15 min at 4°C, the reaction (100 µl total volume) was initiated by addition of PGH_2_ at the indicated concentrations (routinely: 20 µM). After 1 min at 4°C, the reaction was terminated using stop solution (100 µl; 40 mM FeCl_2_, 80 mM citric acid, and 10 µM 11β-PGE_2_ as internal standard). Routinely, PGE_2_ was separated by solid-phase extraction and analyzed by RP-HPLC as described previously; MK886 was used as reference inhibitor (Koeberle et al., 2008). In experiments, where 1 and 5 μM PGH_2_ was used as substrate, PGE_2_ was quantified by PGE_2_ High Sensitivity EIA Kit (Abcam, Cambridge, UK) according to the manufacturer’s protocol.

### Determination of the Biosynthesis of COX-Derived Prostanoids in LPS-Activated Human Monocytes

Freshly isolated primary human monocytes (10^6^/ml) were preincubated with GA, indomethacin, or vehicle (0.1% DMSO) at 37°C, and, after 15 min, LPS (100 ng/ml) was added for 24 h. Cells were placed on ice and eicosanoids were extracted as previously described (Werz et al., 1998) and analyzed by ultra-performance liquid chromatography (UPLC)-coupled ESI tandem mass spectrometry (MS/MS). In brief, LMs were separated on an Acquity UPLC BEH C18 column (1.7 µm, 2.1 × 50 mm, Waters, Milford, MA) using an Acquity^™^ UPLC system (Waters, Milford, MA, USA), and chromatography was performed at a flow rate of 0.8 ml/min and a column temperature of 45°C. The solvents for the mobile phase were acetonitrile (A) and water/acetonitrile (90/10; B) both acidified with 0.07% (v/v) formic acid. Isocratic elution at A/B = 30/70 was performed for 2 min and followed by a linear gradient to A/B = 70/30 within 5 min. The chromatography system was coupled to a QTRAP 5500 Mass Spectrometer (AB Sciex, Darmstadt, Germany) equipped with an electrospray ionization source. Parameters were adjusted as previously described (Schaible et al., 2013a). Identification of eicosanoids was based on the detection of specific fragment ions through multiple reaction monitoring. Automatic peak integration was performed with Analyst 1.6 software (AB Sciex, Darmstadt, Germany) using IntelliQuan default settings. Data were normalized on the internal standard PGB_1_ and are given as relative intensities.

### Incubations of Macrophages and LM Metabololipidomics

Macrophages (2 × 10^6^/ml) were incubated in PBS containing 1 mM CaCl_2_. GA or vehicle control (0.1% DMSO) was applied 10 min prior to stimulation with *Escherichia coli* (serotype O6:K2:H1) at a ratio of 1:50 (M1/M2:*E. coli*) for 180 min at 37°C. Supernatants were transferred to 2 ml of ice-cold methanol containing 10 µl of deuterium-labeled internal standards (200 nM d_8_-5*S*-HETE, d_4_-LTB_4_, d_5_-LXA_4_, d_5_-RvD2, and d_4_-PGE_2_ and 10 µM d_8_-AA; Cayman Chemical/Biomol GmbH, Hamburg, Germany) to facilitate quantification. Sample preparation was conducted as described previously (Werner et al., 2019). In brief, samples were kept at −20°C for 60 min to allow protein precipitation. After centrifugation (1200 × *g*, 4°C, 10 min) 8 ml of acidified H_2_O (pH 3.5) was added and subjected to solid-phase extraction. Solid-phase cartridges (Sep-Pak^®^ Vac 6cc 500 mg/6 ml C18; Waters, Milford, MA) were equilibrated with 6 ml of methanol and 2 ml of H_2_O before samples were loaded onto columns. After washing with 6 ml of H_2_O and additional 6 ml of *n*-hexane, LMs were eluted with 6 ml of methyl formate. Finally, the samples were brought to dryness using an evaporation system (TurboVap LV, Biotage, Uppsala, Sweden) and resuspended in 100 µl methanol–water (50/50, v/v) for UPLC-MS/MS automated injections. LM profiling was analyzed with an Acquity™ UPLC system (Waters, Milford, MA) and a QTRAP 5500 mass spectrometer (AB Sciex, Darmstadt, Germany) equipped with a Turbo V™ Source and electrospray ionization (ESI). LMs were eluted using an ACQUITY UPLC^®^ BEH C18 column (1.7 µm, 2.1 × 100 mm; Waters, Eschborn, Germany) as reported before (Werner et al., 2019). The QTRAP 5500 mass spectrometer was operated in negative ionization mode using scheduled multiple reaction monitoring (MRM) coupled with information-dependent acquisition. The scheduled MRM window was 60 s, optimized LM parameters were adopted, and the curtain gas pressure was set to 35 psi. The retention time and at least six diagnostic ions for each LM were confirmed by means of an external standard (Cayman Chemicals). Quantification was achieved by calibration curves for each LM. Linear calibration curves were obtained for each LM and gave *r* ^2^ values of 0.998 or higher (for fatty acids 0.95 or higher). Additionally, the limit of detection for each targeted LM was determined.

### Determination of the COX-1-Derived Product 12-HHT in Human Platelets

Freshly isolated human platelets (1 × 10^8^/ml PGC buffer) were pre-incubated with the test compound for 15 min at 37°C and stimulated for 10 min at 37°C with 5 µM AA. The COX reaction was stopped after 10 min at 37°C by the addition of 1 ml of ice-cold methanol, and the formed 12-HHT was analyzed by HPLC as previously described (Siemoneit et al., 2008).

### Activity Assays of Isolated COX-1 and COX-2

Inhibition of ovine COX-1 and human COX-2 was investigated as previously described (Siemoneit et al., 2008). Briefly, purified ovine COX-1 (50 units, Cayman Chemical, Ann Arbor, MI) or human recombinant COX-2 (20 units, Cayman Chemical, Ann Arbor, MI) were diluted in 1 ml of reaction mixture containing 100 mM Tris buffer pH 8, 5 mM glutathione, 5 µM hemoglobin, and 100 µM EDTA at 4°C and pre-incubated with the test compound for 5 min. Samples were pre-warmed for 60 s at 37°C, and AA (5 µM for COX-1 and 2 µM for COX-2) was added to start the reaction. After 5 min at 37°C, the reaction was stopped, PGB_1_ as standard was added and 12-HHT was extracted and then analyzed by HPLC (Siemoneit et al., 2008). Indomethacin (10 µM) was used as a well-recognized reference inhibitor of COX-1 and celecoxib for COX-2 to control the assays.

### Determination of the Activity of Isolated Human Recombinant cPLA_2_α

The cPLA_2_α coding sequence was cloned from pVL1393 plasmid (kindly provided by Dr. Wonhwa Cho, University of Illinois at Chicago) into pFastBacTM HT A containing a 6× his-tag coding sequence. The recombinant plasmid was transformed into DH10BacTM *E. coli*. Sf9 cells were transfected with recombinant bacmid DNA using Cellfectin^®^ Reagent and the generated baculovirus was amplified. Overexpression of His-tagged cPLA_2_ in baculovirus-infected Sf9 cells and isolation using Ni-NTA agarose beads was performed as previously described (Hoffmann et al., 2010). Multilamellar vesicles (MLVs) were prepared by drying 1-palmitoyl-2-arachidonyl-*sn*-glycero-3-phosphocholine (PAPC) (Avanti Polar Lipids, Inc., Alabaster, AL) and 1-palmitoyl-2-oleoyl-*sn*-glycerol (POG) (Avanti Polar Lipids, Inc., Alabaster, AL) in a ratio of 2:1 (nmol:nmol, in chloroform) under nitrogen in glass vials. After the addition of 20 mM Tris buffer (pH 7.4) containing 134 mM NaCl and 1 mg/ml fatty acid-free BSA, the MLV suspension was disrupted by several freeze–thaw cycles (liquid nitrogen) and then extruded 11 times with a mini-extruder (Avanti Polar Lipids, Inc., Alabaster, AL) through a polycarbonate membrane (100 nm pore diameter) at room temperature (above transition temperature of the lipids) to produce large unilamellar vesicles (LUVs). Final lipid concentrations were in total 250 µM in 200 µl. Test compounds and 1 mM CaCl_2_ were added to the vesicles, and the reaction was started by the addition of 500 ng of his-tagged cPLA_2_ (in 10 µl buffer). After 1 h at 37°C, 1.6 ml of methanol was added, and AA was extracted by RP-18 solid-phase extraction. Following derivatization with p-anisidinium chloride, the resulting derivate was analyzed by RP-HPLC at 249 nm as previously described (Hoffmann et al., 2010).

### DPPH Assay

The radical scavenger capability was assessed by measuring the reduction of the stable free radical 2,2-diphenyl-1-picrylhydrazyl (DPPH) as previously described (Schaible et al., 2013b). Briefly, up to 100 µM GA was tested and added to a final solution of 200 µl containing the stable free radical DPPH in ethanol (50 µM, corresponding to 5 nmol), buffered with acetate to pH 5.5, in a 96-well plate. The absorbance was recorded at 520 nm (Multiskan Spectrum Reader, Thermo Fisher Scientific Oy, Vantaa, Finland) after 30 min of incubation under gentle shaking in the dark. Ascorbic acid and *L*-cysteine were used as reference compounds. All analyses were performed in triplicate.

### Pharmacophore-Based Virtual Screening

The used virtual 3D molecular CHM database comprising 10,216 unique compounds was generated as described previously (Fakhrudin et al., 2010). The database was virtually screened with two earlier reported pharmacophore models for mPGES-1 inhibitors (Waltenberger et al., 2011) using the “Search 3D database” protocol of Discovery Studio 2.0 (BIOVIA, San Diego, CA, USA) in FAST mode.

### Docking Simulation

GA was docked into the binding site of the mPGES-1 crystal structure [Protein Data Bank (PDB) entry 4bpm], a trimer co-crystallized with glutathione (GSH) and the ligand 2-[[2,6-bis(chloranyl)-3-[(2,2-dimethylpropanoylamino)methyl]phenyl]amino]-1-methyl-6-(2-methyl-2-oxidanyl-propoxy)-*N*-[2,2,2-tris(fluoranyl)ethyl]benzimidazole-5-carboxamide (*K*_I_ = 2.4 nM) (Li et al., 2014). The binding site was defined at the location of the co-crystallized ligand with a radius of 8 Å. GOLD v.5.2 (Jones et al., 1997) was used. CHEMPLP was selected as a scoring function to evaluate the quality of the individual poses. The best ranked pose had a score of 74.53.

Molecular docking of GA into 5-LO was performed using GOLD 5.2. The crystal structure 3O8Y (Gilbert et al., 2011) was prepared by inserting four virtual mutations (mutated residues Glu13, His14, Gly75, and Ser76 in the stable 5-LO to Trp13, Phe14, Trp75, and Leu76 in the wild-type enzyme) to represent the wild-type enzyme rather than the crystallized stable 5-LO (Gilbert et al., 2011). It was then energetically minimized in Discovery Studio (version 3.5). CHEMPLP was selected as a scoring function to evaluate the quality of the individual poses. The binding site was defined between the catalytic domain and the C2 domain around Ile167 in a 10-Å radius.

### SDS-Page and Western Blot

Cell lysates of monocytes (2 × 10^6^ cells) were separated on 10% polyacrylamide gels and blotted onto nitrocellulose membranes (Amersham^™^ Protran Supported 0.45 µm nitrocellulose, GE Healthcare, Freiburg, Germany). The membranes were incubated with the following primary antibodies: rabbit polyclonal anti-COX-2, 1:500 (4842, Cell Signaling) and rabbit polyclonal anti-β-actin, 1:1,000 (4967S, Cell Signaling). Immunoreactive bands were stained with IRDye 800CW Goat anti-Rabbit IgG (H+L), 1:15,000 (926 32211, LI-COR Biosciences), and visualized by an Odyssey infrared imager (LI-COR Biosciences, Lincoln, NE).

### Statistical Analysis

Data are expressed as mean ± S.E.M. IC_50_ values were calculated from averaged measurements at five different concentrations of the compounds by nonlinear regression using GraphPad Prism4 software (San Diego, CA) one site binding competition. Statistical evaluation of the data was performed by one-way ANOVA followed by a Bonferroni or Tukey–Kramer *post hoc* test for multiple comparisons, respectively. If indicated, data were log-transformed to generate stronger Gaussian-distributed data sets amenable to parametric analysis. Paired *t* test was used for comparison of two groups. The criterion for statistical significance is *P* < 0.05.

## Results

### Identification of GA as a Potential mPGES-1 Inhibitor by Pharmacophore-Based Virtual Screening

**Figure 1:**
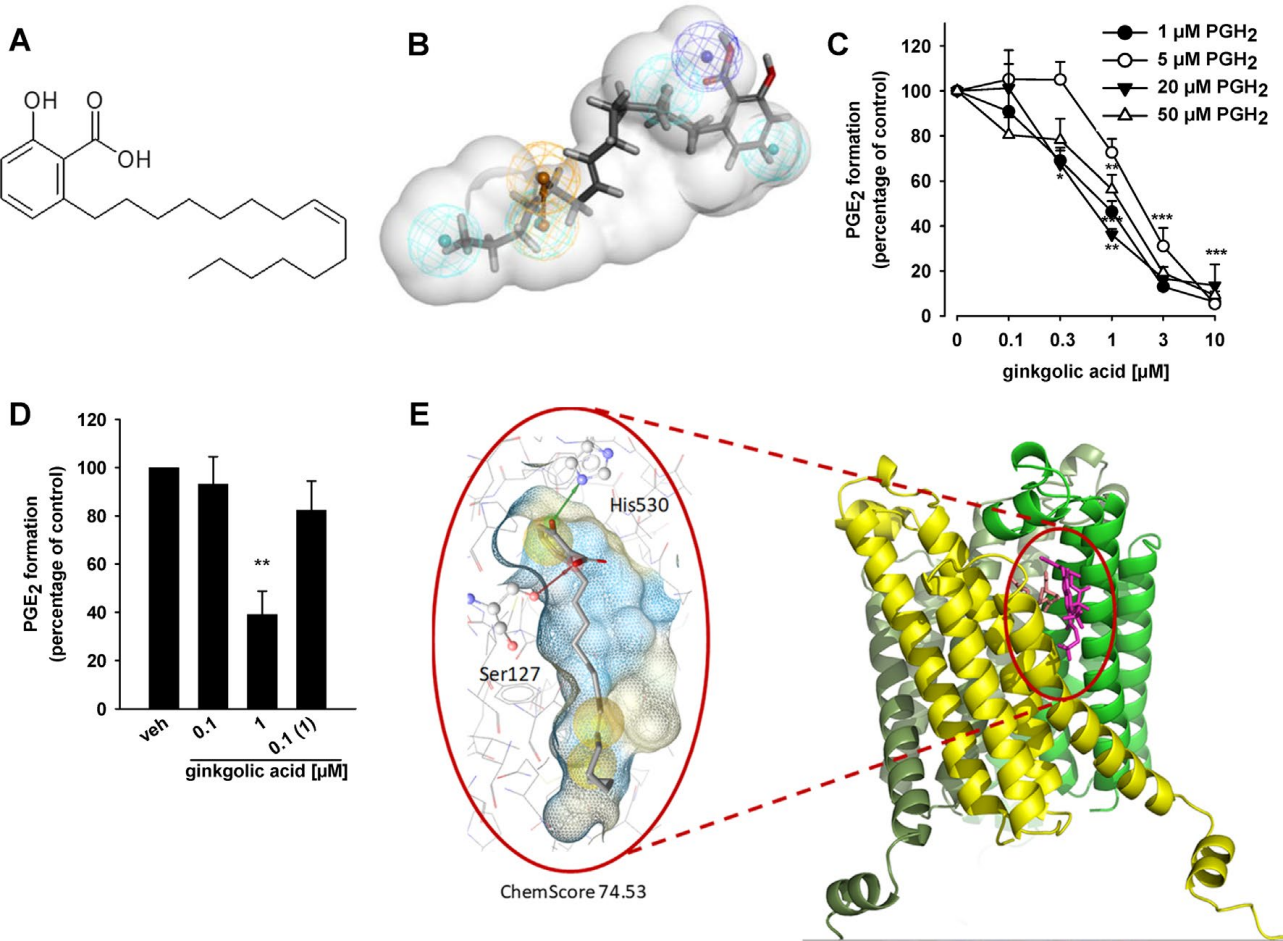
Ginkgolic acid (GA) targets mPGES-1 activity. **(A)** Chemical structure of ginkgolic acid (GA). **(B)** GA mapped to the pharmacophore model M2 for acidic inhibitors of mPGES-1 (Bauer et al., 2012). It mapped the negatively ionizable feature (dark blue) and the four hydrophobic features (cyan), while the aromatic ring feature (brown) was omitted. **(C)** Inhibition of mPGES-1 activity; concentration–response analysis. Microsomal preparations of IL-1β-stimulated A549 cells were pre-incubated with GA or vehicle (0.3% DMSO) for 15 min at 4°C and PGE_2_ formation was induced by addition of PGH_2_ at 1, 5, 20, or 50 µM, as indicated. The amount of PGE_2_ was quantified for 1 and 5 µM PGH_2_ by the use of a PGE_2_ High Sensitivity EIA Kit (Abcam, Cambridge, UK) according to the manufacturer’s protocol, and PGE_2_ formation of samples incubated with 20 and 50 µM PGH_2_ was analyzed by RP-HPLC. Data are given as mean ± S.E., *n* = 3, *p < 0.05, **p < 0.01, ***p < 0.001 vs. vehicle (DMSO) control, ANOVA + Tukey HSD *post hoc* tests. **(D)** Reversibility of mPGES-1 inhibition. Microsomal preparations were pre-incubated for 15 min with 1 µM GA or vehicle (DMSO), and an aliquot was diluted 10-fold to obtain an inhibitor concentration of 0.1 µM. Then, 20 µM PGH_2_ was added and all samples were incubated for 1 min on ice, and PGE_2_ formation was analyzed as described by RP-HPLC. Data are given as mean ± S.E., *n* = 3, **p < 0.01 vs. vehicle (DMSO) control, ANOVA + Tukey HSD *post hoc* tests. **(E)** GA fitted into the binding site of mPGES-1, located between two monomer units (yellow and green), in a molecular docking simulation. The chemical interactions are color-coded: yellow spheres, hydrophobic; red arrow, hydrogen bond acceptor; green arrow, hydrogen bond donor. The surface is colored by aggregated hydrophilicity (blue)/hydrophobicity (gray). Amino acids participating in hydrophilic interactions are depicted in ball-and-stick style.

Aiming at identifying novel natural products that inhibit mPGES-1, our in-house CHM database comprising 10,216 compounds that are reported as ingredients of medicinal preparations used in the traditional Chinese medicine (TCM) (Fakhrudin et al., 2010) was virtually screened using two established pharmacophore models for mPGES-1 inhibitors (Waltenberger et al., 2011). Model M1 consists of one negatively ionizable feature, four hydrophobic features, one aromatic ring feature, and a shape restriction with a maximum extent ratio set as 1.3 limiting the size of fitting virtual hits. M2, the partial query model of M1, allows either the aromatic feature or one of the hydrophobic features to be omitted and thus, recognizes more chemically diverse mPGES-1 inhibitors (Waltenberger et al., 2011). Virtual screening of the CHM database with M1 achieved a hit rate of only 0.04% (4 molecules), while virtual screening with the less restrictive pharmacophore model M2 led to a hit rate of 0.6% (61 molecules). Over 10% of the obtained virtual hits were identified as depsides from lichen species (Bauer et al., 2012); more detailed information is given in the Supporting Information. Two biologically active compounds, that is, the depsides physodic acid and olivetoric acid recognized by M2 within another virtual screening approach, did not map the aromatic ring feature, suggesting that this feature may not be important for mPGES-1 inhibition (Bauer et al., 2012). The hit list from the virtual screening of the CHM database with M2 comprised GA, which also did not map the aromatic ring feature and which has a chemical scaffold that differs significantly from those of the depsides (**Figure 1A and B**). GA is a prominent ingredient of *G. biloba*, and thus we selected the compound for biological testing and investigation of its pharmacological profile and molecular mechanism.

### mPGES-1 is a Direct Functional Target of GA

To confirm mPGES-1 as a target of GA, we studied the ability of the compound to inhibit the mPGES-1-mediated formation of PGE_2_ from exogenous PGH_2_ in a cell-free assay using microsomes from IL-1β-stimulated A549 cells as mPGES-1 source. GA (10 µM) suppressed mPGES-1-mediated conversion of PGH_2_ to PGE_2_ by 89 ± 8%, which is comparable to the reference inhibitor MK886 (83 ± 6%, not shown) that inhibits human mPGES-1 with an IC_50_ of 2.4 µM (Koeberle et al., 2008). More detailed concentration response studies at the standard substrate concentration of 20 µM PGH_2_ revealed an IC_50_ of 0.7 µM for suppression of mPGES-1 (**Figure 1C**). Alteration of PGH_2_ to lower (1 and 5 µM) or higher (50 µM) concentrations led to only slight changes in the inhibitory potency, suggesting that the efficiency of GA to inhibit mPGES-1 is independent of the substrate concentration (**Figure 1C**). In addition, a reversible mechanism of mPGES-1 is apparent, as GA failed to efficiently block mPGES-1 derived PGE_2_ formation after washout (10-fold dilution) (**Figure 1D**).

To analyze the putative binding mode of GA in mPGES-1, the compound was fitted into the binding site of the mPGES-1 crystal structure (Li et al., 2014), downloaded from the PDB (Berman et al., 2000), in a molecular docking simulation. In the preferred docking poses, the carboxyl group of GA interacted with Ser127, while the hydroxyl group formed a hydrogen bond with His530 (**Figure 1E**). The hydrophobic chain is located in a grove on the surface of mPGES-1 (**Figure 1E**).

### Effects of GA on COX and TXA Synthase Activities

**Figure 2:**
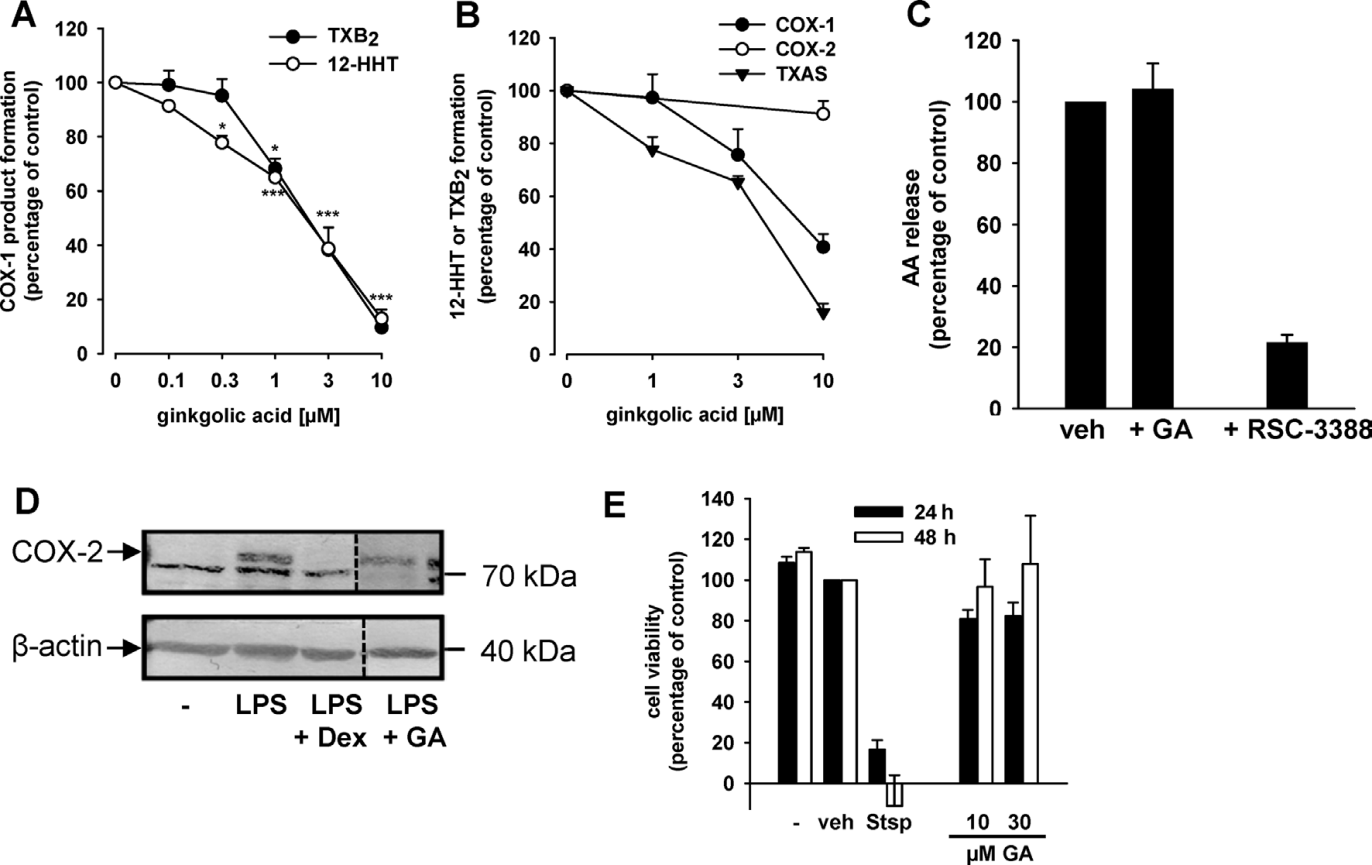
GA inhibits COX-1 and TXAS activities without affecting COX-2, cPLA_2_, and cell viability. **(A)** GA inhibits COX-1-dependent TXB_2_ and 12-HHT formation in human platelets. Platelets (10^8^/ml PBS pH 7.4 containing 1 mM CaCl_2_) were pre-incubated with GA or vehicle (0.3% DMSO) for 5 min prior to stimulation with AA (5 µM). After another 5 min at 37°C, 12-HHT was determined by RP-HPLC and TXB_2_ using a TXB_2_ High Sensitivity EIA Kit. Data are means + S.E., *n* = 3, *p < 0.05, ***p < 0.001 vs. vehicle (DMSO) control (=100%), ANOVA + Tukey HSD *post hoc* tests. **(B)** Purified ovine COX-1 (50 units) or human recombinant COX-2 (20 units) were pre-incubated with GA for 5 min prior to stimulation with AA (5 µM or 2 µM, respectively). After 10 min, 12-HHT formation was analyzed using RP-HPLC. TXAS activity was determined in platelet homogenates (10^8^/ml PBS containing 1 mM CaCl_2_, sonified 3 × 15 s) pre-incubated for 15 min with GA on ice and stimulated with 20 µM PGH_2_ for 1 min. TXB_2_ levels were assessed using a TXB_2_ High Sensitivity EIA Kit. Data are means + S.E., *n* = 3–4, **p < 0.01, ***p < 0.001 vs. vehicle (DMSO) control, ANOVA + Tukey HSD *post hoc* tests. **(C)** Effects of GA (10 µM) and RSC-3388 (1 µM) on cPLA_2_ in a cell-free assay. Freshly prepared large unilamellar vesicles were incubated with cPLA_2_ enzyme, 1 mM Ca^2+^, and the indicated compounds for 1 h at 37°C. After derivatization with p-anisidinium chloride, the resulting derivate was analyzed by RP-HPLC. Data are means + S.E., *n* = 3, ***p < 0.001 vs. vehicle (DMSO) control, ANOVA + Tukey HSD *post hoc* tests. **(D)** Expression of COX-2 protein. Human monocytes were preincubated with GA (10 µM), dexamethasone (1 µM), or vehicle (0.1% DMSO), and then stimulated with 1 µg/ml lipopolysaccharide (LPS) for 24 h at 37°C. Cells were detached and lysed. Samples were subjected to SDS-PAGE and Western blot using a specific antibody against COX-2 (upper band, indicated by arrow); β-actin was used as loading control. Results are representative of three independent experiments. **(E)** Cytotoxicity in A549 cells. Cells were incubated for 24 h and 48 h at 37°C with GA, staurosporine (Stsp, 3 µM), or vehicle (0.1% DMSO). Then, MTT was added and samples were incubated for 4 h at 37°C. The reaction was stopped and the absorption was measured at 570 nm. Cell viability is reported as percentage of vehicle control. Means + S.E. of *n* = 3.

In order to investigate if GA could also interfere with other enzymes involved in the biosynthesis of PGE_2_, we analyzed its ability to interfere with the activities of COX-1 and COX-2 in cell-based and cell-free assays. The formation of the COX-1 products 12-HHT and TXB_2_ was analyzed in human platelets stimulated with 3 µM AA as a substrate of COX-1. GA concentration-dependently blocked 12-HHT and TXB_2_ formation with similar IC_50_ values of 2.1 and 2.2 µM, respectively (**Figure 2A**). We tested whether the effect of GA in the cell-based assay is due to direct interference with COX-1. For this purpose, isolated ovine COX-1 was used as enzyme source and 5 µM AA as substrate; the COX-1 inhibitor indomethacin was used as control. Whereas indomethacin completely blocked COX product formation at 10 µM as expected (not shown), GA moderately inhibited 12-HHT formation by isolated COX-1 with IC_50_ = 8.1 µM (**Figure 2B**). In contrast, GA failed to inhibit the activity of isolated human recombinant COX-2 in a cell-free assay using 2 µM AA as substrate (**Figure 2B**), whereas celecoxib (5 µM, used as reference drug) strongly blocked COX-2 activity (not shown).

Besides inhibition of COX-1, suppression of TXAS might be causative for reduced 12-HHT and TXB_2_ levels in intact platelets as well (Shen and Tai, 1986). Thus, we studied the effects of GA on TXAS-derived TXB_2_ from exogenously added PGH_2_ (thus circumventing COX-1 activity) in platelet homogenates. The TXAS inhibitor CV4151 (1 µM, used as reference drug) completely blocked TXB_2_ formation in this assay as expected (not shown). Similar as found for COX-1, GA also inhibited TXAS activity in a concentration-dependent manner with IC_50_ = 5.2 µM (**Figure 2B**). Finally, we studied the effects of GA on the enzymatic activity of human recombinant cPLA_2_-α, the enzyme providing free AA as substrate for COX-1/2 in prostanoid biosynthesis. While the cPLA_2_-α inhibitor RSC-3388 (1 µM) effectively blocked AA release from phospholipid micelles in a cell-free assay, GA (10 µM) did not interfere with cPLA_2_-α activity (**Figure 2C**).

Next, the effect of GA on multiple COX-derived prostanoids was assessed using LPS-activated monocytes (24 h), and indomethacin was used as reference COX inhibitor. GA differentially affected the synthesis of COX-derived products formed under these conditions (**Table 1**). Thus, the synthesis of mPGES-1-derived PGE_2_ and of TXAS-derived TXB_2_ and 12-HHT was strongly suppressed, as anticipated, while other prostanoids were less affected (**Table 1**). However, PGF_2α_ formation was significantly reduced, implying modulation of this biosynthetic pathway as well. In contrast, the COX inhibitor indomethacin efficiently blocked the formation of all COX products, irrespective of the terminal prostanoid synthase involved (**Table 1**). The possibility that GA may interfere with the LPS-induced *de novo* synthesis of COX-2 protein can be neglected as GA failed to reduce the expression of COX-2 protein, whereas dexamethasone (1 µM, used as control) blocked COX-2 expression (**Figure 2D**). Moreover, in human monocytes 10 or 30 µM GA, in contrast to 3 µM staurosporine, showed no significant cytotoxic effects after 24 or 48 h of incubation, implying that unspecific detrimental features on cell viability are not relevant for suppression of PGE_2_ and TXB_2_/12-HHT synthesis in the cell-based assays (**Figure 2E**).

**Table 1:** Effect of ginkgolic acid on cyclooxygenase (COX)-derived prostanoid formation by monocytes stimulated with lipopolysaccharide (LPS) for 24 h.

	Ginkgolic acid (GA)	Indomethacin
PGE_2_	40.8 ± 15.1*	3.3 ± 1.2***
PGD_2_	74.0 ± 14.0	35.9 ± 15.2*
TxB_2_	12.4 ± 4.2***	0.7 ± 0.2***
PGF_2α_	24.3 ± 9.5**	5.8 ± 2.4***
11-HETE	52.1 ± 11.8	8.4 ± 2.6*
12-HHT	38.7 ± 8.8*	5.4 ± 1.4**
PGE_1_	84.0 ± 27.3	25.6 ± 9.9**
TxB_1_	77.2 ± 11.0	80.6 ± 9.6

Residual levels of COX-derived prostanoids are calculated as percentage of vehicle (DMSO) control and given as mean ± S.E.M. of single determinations obtained in three independent experiments. *p < 0.05, **p < 0.01, ***p < 0.001; Student unpaired t test.

### GA Inhibits Human 5-LO, the Key Enzyme in Leukotriene Biosynthesis

**Figure 3:**
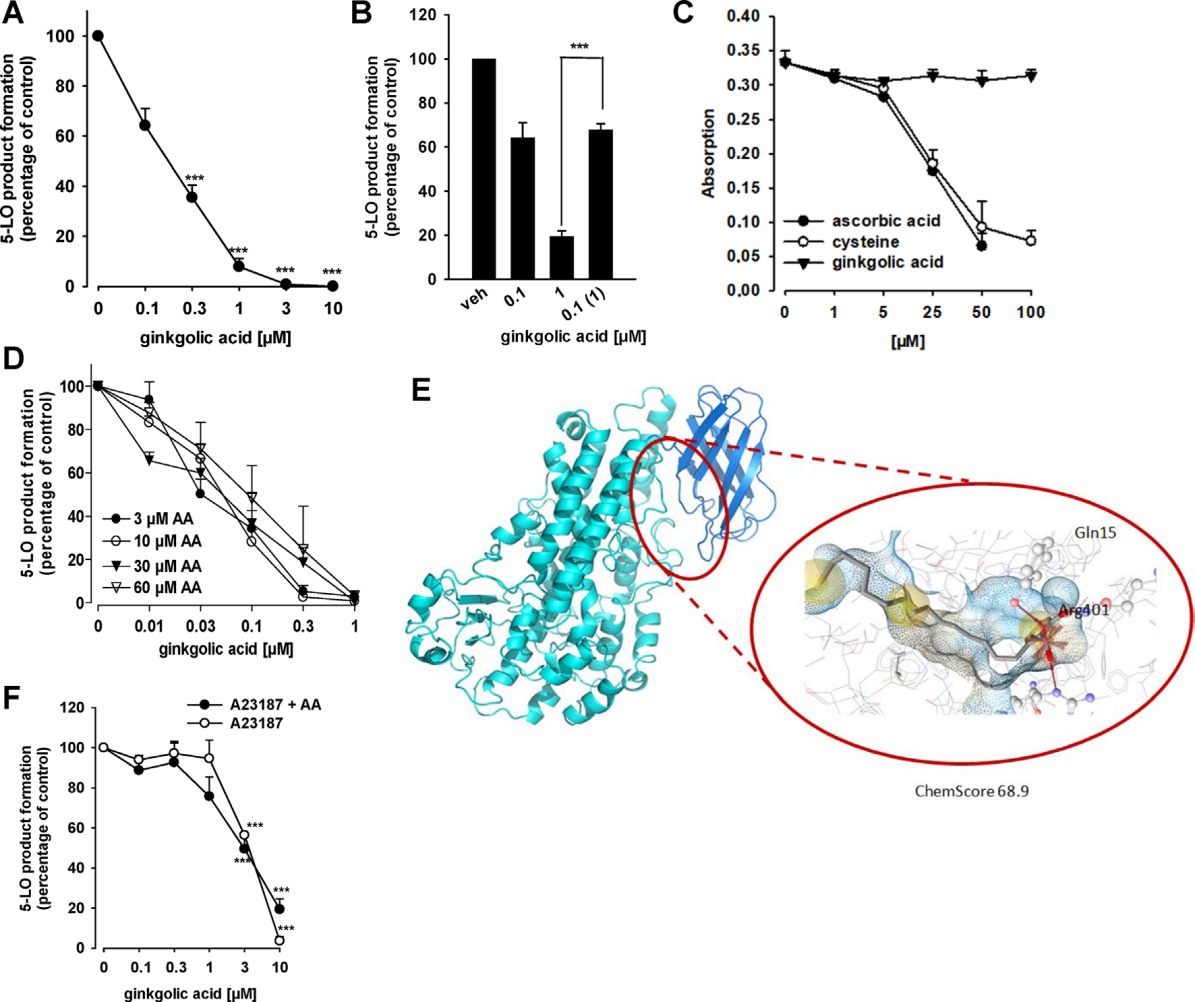
Effects of GA on 5-LO activity. **(A)** Inhibition of isolated 5-LO. Semi-purified human recombinant 5-LO was pre-incubated with GA or vehicle (0.1% DMSO) for 10 min at 4°C. 5-LO product formation was initiated by addition of 20 µM AA for 10 min at 37°C. Then, 5-LO products [tr-LTB_4_ isomers and 5-H(P)ETE] were analyzed by RP-HPLC. Means + S.E.M., *n* = 3, *p < 0.05 and ***p < 0.001 vs. vehicle (DMSO) control, ANOVA + Tukey HSD *post hoc* tests. **(B)** Reversibility of 5-LO inhibition. Semi-purified 5-LO was pre-incubated with 0.1 or 1 µM GA for 10 min at 4°C. One sample containing 1 µM GA was then diluted 10-fold by PBS pH 7.4 to obtain 0.1 µM GA. As control, 10-fold diluted 5-LO in PBS pH 7.4 was pre-incubated with vehicle. 5-LO product formation was initiated by addition of 20 µM AA for 10 min at 37°C. 5-LO products [tr-LTB_4_ isomers and 5-H(P)ETE] were analyzed by RP-HPLC. Means + S.E.M, *n* = 3–4, ***p < 0.001; 1 µM vs. diluted sample, ANOVA + Tukey HSD *post hoc* tests. **(C)** Radical scavenger capability. GA, vehicle and controls were mixed with DPPH solution and incubated for 30 min in the dark. The absorbance was measured at 520 nm. Means + S.E.M., *n* = 3. **(D)** Inhibition of 5-LO by GA at various substrate concentrations. Semi-purified human recombinant 5-LO was pre-incubated with GA or vehicle (DMSO) for 10 min at 4°C. 5-LO product formation was initiated by addition of the indicated concentrations of AA for 10 min at 37°C. 5-LO products [tr-LTB_4_ isomers and 5-H(P)ETE] were analyzed by RP-HPLC. Means + S.E.M., *n* = 3, *p < 0.05 and ***p < 0.001 vs. vehicle (DMSO) control, ANOVA + Tukey HSD *post hoc* tests. **(E)** GA docked into an allosteric binding site of 5-LO, located between the membrane binding domain (dark blue) and the catalytic domain (light blue). Chemical interactions are color-coded: yellow spheres, hydrophobic; red arrows, hydrogen bond acceptor; red star, ionic interaction. The surface is colored by aggregated hydrophilicity (blue)/hydrophobicity (gray). Amino acids participating in hydrophilic interactions are depicted in ball-and-stick style. **(F)** Inhibition of 5-LO product synthesis by GA in intact cells. PMNL (5 × 10^6^/ml) were pre-incubated with GA or vehicle (DMSO) for 10 min at 37°C and stimulated with 2.5 µM A23187 or 2.5 µM A23187 plus 20 µM AA. 5-LO products (LTB_4_, tr-LTB_4_ isomers and 5-HETE) were determined by RP-HPLC. Means ± S.E.M., *n* = 3–5, ***p < 0.001 vs. vehicle (DMSO) control, ANOVA + Tukey HSD *post hoc* tests.

Because many lipophilic acidic molecules that inhibit mPGES-1 often interfere also with 5-LO (Koeberle and Werz, 2015; Koeberle et al., 2016; Koeberle and Werz, 2018), we investigated whether or not GA modulates the 5-LO pathway. In fact, the activity of isolated human recombinant 5-LO in a cell-free assay at a substrate concentration of 20 µM AA was concentration-dependently inhibited by GA with an IC_50_ of 0.2 µM (**Figure 3A**). Zileuton, used as reference 5-LO inhibitor, blocked 5-LO activity as well, with an about threefold higher IC_50_ of 0.6 µM (not shown), which is in agreement with the literature (Carter et al., 1991; Schaible et al., 2013a). Washout experiments demonstrate that inhibition of 5-LO by GA (1 µM) is fully reversible. In fact, the potent suppression of 5-LO was reversed when the enzyme was first incubated with 1 µM GA and then 10-fold diluted in PBS pH 7.4 to achieve a final GA concentration of 0.1 µM (**Figure 3B**).

Several 5-LO inhibitors act by uncoupling the catalytic cycle of 5-LO due to redox properties (Werz and Steinhilber, 2005), and this may apply also to GA. However, antioxidant properties of GA were not obvious, since the compound up to 100 µM failed to reduce the stable DPPH radical, whereas the positive controls ascorbic acid and *L*-cysteine reduced DPPH as expected (**Figure 3C**). Another potential 5-LO inhibitory mechanism could be related to competition with AA as substrate at the active site of 5-LO. However, 5-LO inhibition by GA was consistent at different substrate concentrations ranging from 3 to 60 µM (**Figure 3D**), suggesting that GA may inhibit 5-LO by interference with a distinct site than the AA binding pocket. In fact, molecular docking simulations indicate that GA fits into an allosteric binding site located between the catalytic domain and the C2 domain (**Figure 3E**). In the observed poses, the carboxyl group formed hydrogen bonds with Tyr81 and Arg401, while the hydroxyl group interacted with Gln15 (**Figure 3E**). In this binding site, Gln15 and Tyr81 revealed to be key interaction partners also in other studies (Alsabil et al., 2016; Pein et al., 2018). The described allosteric binding site has also been shown to display similar characteristics as the binding site of mPGES-1 (Cheung et al., 2018), which explains the occurrence of dual 5-LO/m-PGES-1 inhibitors.

Because 5-LO is a tightly regulated enzyme and its inhibition can be manipulated by endogenous cellular factors (e.g., phosphorylation, redox tone) (Werz and Steinhilber, 2005), we analyzed the efficiency of GA to suppress 5-LO product biosynthesis in intact human neutrophils, stimulated with A23187 in the presence and absence of the exogenous substrate AA. As shown in **Figure 3F**, GA inhibited 5-LO product formation independent of the absence or presence of exogenous AA comparably well (IC_50_ = 3.8 and 2.9 µM, respectively), but less potent as compared to isolated 5-LO in the cell-free assay. Zileuton, used as reference inhibitor, suppressed 5-LO product formation in neutrophils (IC_50_ = 1.1 µM) as expected (not shown).

### GA Affects LM Profiles in Bacteria-Activated Human Macrophages

**Figure 4:**
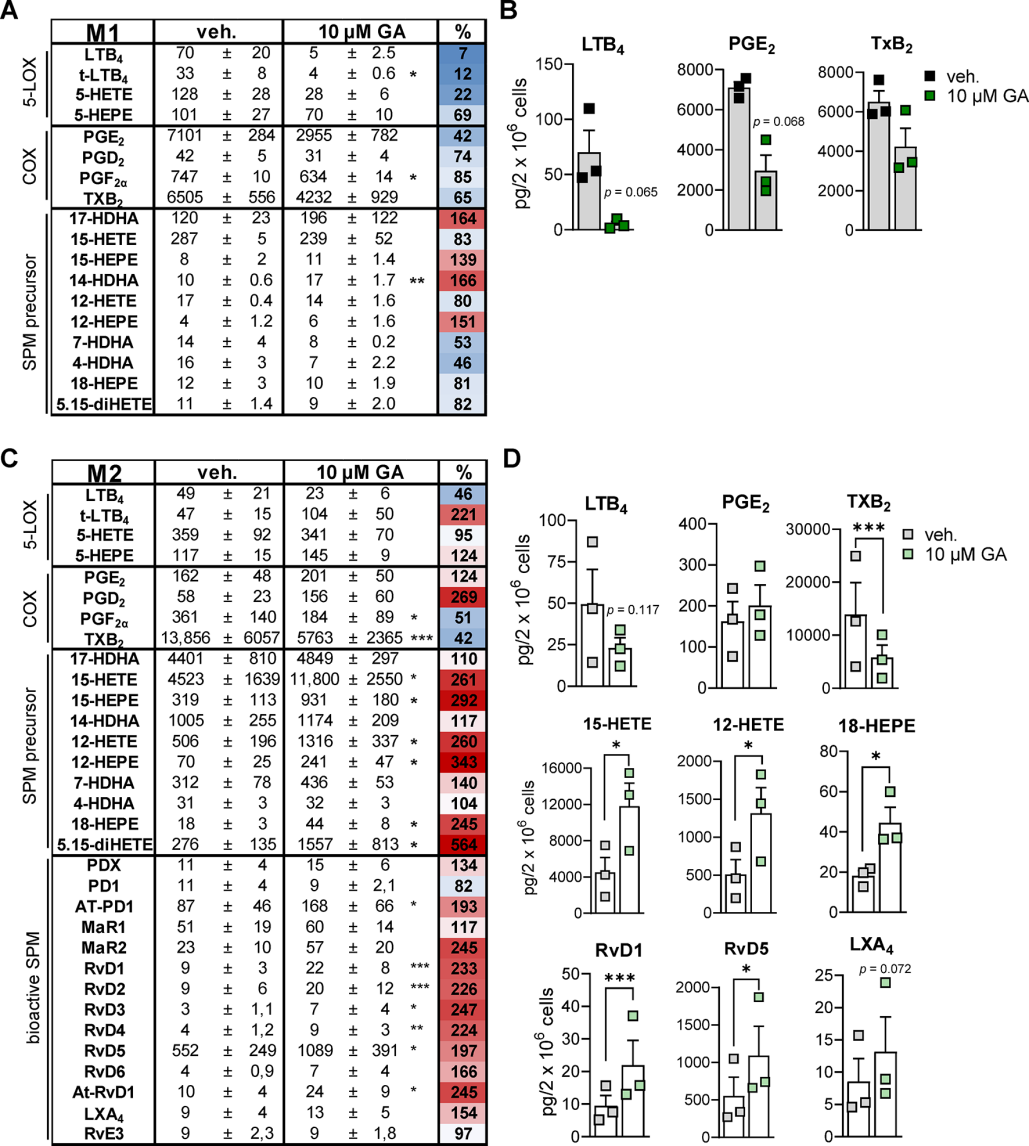
Effects of GA on LM profiles in human M1 and M2 macrophages. Human M1 **(A** and **B)** or M2 **(C** and **D)** macrophages (2 × 10^6^ cells/ml) were preincubated with 10 µM GA or vehicle (0.1% DMSO) for 10 min at 37°C before exposure to pathogenic *E. coli* (O6:K2:H1; ratio 1:50) for 3 h. LMs were extracted by SPE and analyzed by UPLC-MS-MS. **(A** and **C)** Means ± SEM are shown, and the heat map represents the percentage of vehicle-treated cells (=100% control, white; red > 100%; blue < 100%); *n* = 3. **(B)** Effects of GA on LTB_4_, PGE_2_, and TXB_2_ biosynthesis in M1, shown as pg/2 × 10^6^ cells; SPM were below detection limit (<0.5 pg). **(D)** Effects of GA on LTB_4_, PGE_2_, TXB_2_, 15-HETE, 12-HETE, 18-HEPE, RvD1, RvD5, and LXA_4_ biosynthesis in M2, shown as pg/2 × 10^6^ cells. Data were log-transformed for Student’s paired *t* test; *p < 0.05, **p < 0.01, and ***p < 0.001 vs. vehicle (DMSO) control.

To study how the suppressive effects of GA against 5-LO, mPGES-1, and TXAS affects LM biosynthesis in more complex LM networks, we employed a more sophisticated and pathophysiologically relevant cell-based model using human monocyte-derived macrophage subsets (Werner et al., 2019) that represent pro-inflammatory M1- and anti-inflammatory M2-like phenotypes (Motwani and Gilroy, 2015). Upon exposure to pathogenic *E. coli* for 3 h, the M1 phenotype produces mainly PG and LT while M2 macrophages generate predominantly 12/15-LO products including SPM (Werz et al., 2018). Preincubation of M1 with 10 µM GA caused potent suppression of *E. coli*-induced generation of 5-LO products within 3 h, and among the PGs, inhibition of abundantly formed PGE_2_ (7101 pg) was most pronounced (**Figures 4A, B**). Similarly, TXB_2_ formation was also suppressed by GA, particularly in M2 that strongly express TXAS (Gabrielsen et al., 2010), with high TXB_2_ levels (13,856 pg) and, thus, strong TXAS activity (**Figures 4C, D**). These data support a preferential inhibition of 5-LO, mPGES-1, and TXAS by GA. Note that, in M2, the minute PGE_2_ levels (162 pg) were not further impaired by GA (**Figure 4C**), probably because mPGES-1 is less expressed in M2 (Mosca et al., 2007). Of interest, in M2, all 12/15-LO-derived SPM (e.g., RvD1, RvD5, LXA4, **Figures 4C, D**), except PD1 and RvE3, were markedly increased, which correlates to strongly elevated levels of monohydroxy SPM precursors particularly formed from AA and EPA (e.g., 12-HETE, 15-HETE, 12-HEPE, 15-HEPE) (**Figure 4D**). It should be noted that the amounts of free fatty acid substrates (i.e., AA, EPA and DHA) were hardly affected (93–108% versus vehicle control, not shown). In contrast to M2, M1 do not express appreciable amounts of 12/15-LOX and do not form significant SPM levels (Werz et al., 2018; Werner et al., 2019). These data imply that GA effectively inhibits 5-LO-, mPGES-1-, and TXAS-mediated formation of pro-inflammatory LM in human bacteria-stimulated macrophages with a congruent increase of 12/15-LO-derived SPM.

## Discussion

Preparations of *G. biloba* have been used for centuries in TCM to treat pulmonary disorders, bladder inflammation, heart dysfunctions, and skin infections (Mahadevan and Park, 2008), and scientific studies revealed the therapeutic benefit of standardized *G. biloba* extracts (e.g., EGb 761) in the treatment of inflammation-related disorders such as Alzheimer’s disease, cardiovascular disease, cancer, and stress, as well as in memory loss and psychiatric disorders (Mahadevan and Park, 2008; Diamond and Bailey, 2013). GA is considered as a major toxic component in *G. biloba* extracts with allergic (Lepoittevin et al., 1989) and genotoxic effects (Liu and Zeng, 2009; Berg et al., 2015), and therefore, the GA content in commercial *G. biloba* preparations is limited to 5 ppm or less to minimize allergic reactions (Mahadevan and Park, 2008). Accordingly, strong efforts were made to comprehensively evaluate the bioactivities of GA on the cellular and molecular level.

GA is well recognized as an inhibitor of SUMOylation (Fukuda et al., 2009) and experimentally exploited to study the role of SUMOylation in various pathologies (Hamdoun and Efferth, 2017; Rott et al., 2017; Liu et al., 2018; Qiu et al., 2018). It was shown to inhibit SUMOylation with an IC_50_ of 3 µM by direct binding to the SUMO-activating enzyme E1, thereby preventing the formation of the E1-SUMO intermediate (Fukuda et al., 2009). However, whether inhibition of SUMOylation accounts for the manifold bioactivities exerted by GA is unclear. In fact, many different biological properties and targets are described for GA *in vitro* and *in vivo* that, on one hand, may partially explain the cytotoxic and allergic actions of GA, but also suggest beneficial pharmacological features, especially as an anti-cancer agent (Itokawa et al., 1987; Zhou et al., 2010; Baek et al., 2017a; Baek et al., 2017b; Yoon et al., 2018). In particular, DNA strand-breaking (Westendorf and Regan, 2000), activation of protein phosphatase 2C (Ahlemeyer et al., 2001), abrogation of STAT3 signaling (Baek et al., 2017b) and the PI3K/Akt/mTOR pathway (Baek et al., 2017a), transformation of mitochondria and uncoupling of oxidative phosphorylation (Hecker et al., 2002), as well as inhibition of SIRT (Ryckewaert et al., 2014) may account for cytotoxic and anti-cancer properties of GA.

Here, we show that GA directly targets human mPGES-1 and 5-LO with high affinities, reflected by the fairly low IC_50_ values of only 0.7 and 0.2 µM, compared to the IC_50_ of 3 µM for interference with SUMOylation (Fukuda et al., 2009) and, for example, 50 µM for induction of phosphatase PTEN and protein tyrosine phosphatase SHP-1 (Baek et al., 2017b) or 100 µM for activation of protein phosphatase 2C (Ahlemeyer et al., 2001). Additional direct targets of GA are COX-1 and TXAS, albeit with lower affinities given the higher IC_50_ values of 8.1 and 5.2, respectively, in cell-free assays, but nevertheless with pharmacologically relevant consequences for prostanoid formation in human platelets and macrophages.

GA was here identified as a potential mPGES-1 inhibitor in an unbiased manner by virtual screening of 10,216 compounds from our in-house CHM database using two previously established pharmacophore models for mPGES-1 inhibitors (Waltenberger et al., 2011). Since many lipophilic acidic natural compounds that inhibit mPGES-1 also act on 5-LO (Koeberle and Werz, 2018), it was conceivable that GA may also interfere with 5-LO catalysis. Our data show that inhibition of both mPGES-1 and 5-LO by GA is independent of the respective substrate concentration and fully reversible, supported by molecular docking simulation that propose concrete binding sites for GA in both enzymes. Similar binding modes as for GA were observed already for several other mPGES-1 inhibitors like depsides and depsidones from lichen (Bauer et al., 2012). As reported previously (Bauer et al., 2012), many of the virtual hits retrieved by these models belong to the chemical class of depsides from lichen, i.e., perlatolic acid (M1), physodic acid (M2), and olivetoric acid (M2). Like for physodic acid and olivetoric acid, which did not fit the aromatic ring feature in M2 (Bauer et al., 2012), the aromatic ring feature was omitted also for GA, suggesting that this feature is negligible. In 5-LO, GA accommodated a site between the catalytic and membrane binding domain of the enzyme, recently proposed to bind a 5-LO inhibitory synthetic *N*-phenylbenzenesulfonamide involving the same residues, i.e., Tyr81 and Arg401 (Cheung et al., 2018).

mPGES-1-derived PGE_2_ plays a crucial role not only in inflammation but also for tumor progression, vascularization, and metastasis (Koeberle and Werz, 2009; Larsson and Jakobsson, 2015). Elevated levels of pro-inflammatory PGE_2_ accompanied by mPGES-1 overexpression were shown to be hallmarks in prostate, breast, and lung cancer cells (Koeberle and Werz, 2009) whereas genetic deletion of the mPGES-1 was beneficial in cancer chemoprevention (Nakanishi et al., 2008). Thus, inhibition of mPGES-1 by GA might in fact contribute to the anti-carcinogenic and anti-inflammatory activities observed by others before (Itokawa et al., 1987; Zhou et al., 2010; Baek et al., 2017a). GA efficiently suppressed PGE_2_ formation in the cellular context, that is, under short-term (3 h) treatment of bacteria-activated M1 macrophages and under long-term (24 h) treatment in LPS-stimulated human monocytes without affecting the cell viability of monocytes. Our data rather exclude that GA may affect cellular PGE_2_ levels due to interference with other PGE_2_-biosynthetic enzymes, since the activity or expression of inducible COX-2 was not affected, and COX-1 (which is inhibited by GA) plays only a minor role in LPS-induced PGE_2_ formation in monocytes (Patrignani et al., 1996). In contrast, the suppressive effects of GA on 12-HHT and TxB_2_ biosynthesis in human platelets might be due to inhibition of COX-1 and to interference with TXAS, since direct interference of GA with both COX-1 and TXAS was evident in cell-free assays. In platelets, 12-HHT is enzymatically formed through TXAS that is abundantly expressed in these cells and specifically catalyzes the conversion of PGH_2_ to 12-HHT (Shen and Tai, 1986). Therefore, whether TXAS or COX-1 is the preferred target of GA in platelets cannot be answered at this stage. In M2 macrophages, where TXAS is strongly expressed (Gabrielsen et al., 2010), TXB_2_ was by far the most abundant prostanoid and its strong formation was effectively suppressed by GA. In contrast, the low levels of PGE_2_ in M2 were unaffected by GA, seemingly due to the fact that mPGES-1 is essentially absent in human M2 macrophages (Mosca et al., 2007).

In comparison to other well-recognized naturally occurring 5-LO inhibitors (Werz, 2007), GA (IC_50_ = 0.2 μM) is highly potent. Like mPGES-1 inhibition, GA targeted 5-LO activity independent of the substrate concentration and inhibition of 5-LO is reversible as seen for many other direct 5-LO inhibitors (Werz et al., 2017). Note that GA did not possess any radical scavenging properties and should thus not act on 5-LO by keeping the non-heme active-site iron in the inactive, reduced state or by interrupting its redox cycle during catalysis, which is the case for many natural product-derived 5-LO inhibitors (Koeberle and Werz, 2018). In intact neutrophils, the efficiency of GA to inhibit 5-LO was 10-fold lower as in cell-free assays, which might be due to an impeded intracellular distribution of GA where 5-LO product biosynthesis takes place (i.e., in complex with FLAP at the nuclear membrane) (Garscha et al., 2016; Gerstmeier et al., 2016). Since GA failed to inhibit the activity of cPLA_2_-α, an enzyme that is key to provide efficient amounts of free AA as substrate for eicosanoid biosynthesis (Leslie, 2015), suppression of PGE_2_ and LT formation at this level can be excluded. In fact, release of AA, EPA, and DHA in *E. coli*-stimulated macrophages was not affected by GA. Along these lines, supplementation of exogenous AA failed to overcome the suppression of 5-LO product formation by GA in neutrophils.

LM biosynthesis inhibitors are commonly evaluated in cellular test systems with limited read-out where mainly inflammation-promoting PG and/or LT in pro-inflammatory leukocytes are addressed. Here, we studied also how GA modulates the overall LM networks in human *E. coli*-stimulated pro-inflammatory M1 that produce mainly PG and LT but also in anti-inflammatory M2 macrophages that generate predominantly SPM (Werz et al., 2018; Werner et al., 2019). These experimental models also allow one to address the inhibitor-induced redirection of substrates to other enzymatic LM pathways (Werner et al., 2019). Our data with these macrophages indicate that GA i) inhibits mPGES-1 in M1; ii) inhibits 5-LO, particularly in M1; iii) inhibits TXAS, particularly in M2; and iv) stimulates 12/15-LO pathways in M2 leading to elevated SPM levels. Whether GA directly stimulates 12/15-LO activities or governs activating regulatory processes (e.g., phosphorylation, subcellular redistribution, etc.) or whether it redirects substrate fatty acids from PG and LT biosynthesis towards SPM remains to be investigated. Nevertheless, since SPMs are highly bioactive LMs that, in contrast to pro-inflammatory PGs and LTs, actively terminate inflammation and promote resolution and tissue repair (Serhan, 2014), the molecular profile of GA targeting multiple LM biosynthetic enzymes might be a valuable prototype for future development of novel anti-inflammatory/pro-resolving drugs.

## Conclusions

In summary, we identified human mPGES-1 and 5-LO as two novel high-affinity targets for GA, and we provide significant evidence for their functional modulation by GA in the cellular context. As PGE_2_ and LTs are both crucial mediators in the development of inflammation-associated cancer, our findings suggest that the anti-cancer properties of GA might be related, at least in part, to downregulation of these eicosanoids in tumor-associated innate immune cells such as M2-like macrophages and neutrophils. The concomitant increase of SPM formation in M2-like macrophages might further promote the anti-tumor properties of GA, as SPMs in fact suppress tumor growth (Sulciner et al., 2018) and stimulate resolution in cancer (Gilligan et al., 2019). Although the usefulness of GA as drug in humans is questionable given its allergic and genotoxic potential, GA, based on its favorable multiple-target-inhibitor profile, might be a tool compound for accomplishing novel pharmacological strategies.

## Data Availability

The raw data supporting the conclusions of this manuscript will be made available by the authors, without undue reservation, to any qualified researcher.

## Author Contributions

Conceived and designed the experiments: JG, JR, HS, AK, DS, and OW. Supervised the experiments: JR, HS, DS, and OW.

Performed the experiments: JG, JS, FW, BW, and VT. Analyzed the data: JG, JS, FW, BW, and VT. Interpreted the data: JG, VT, JR, HS, AK, DS, and OW. Wrote the manuscript: OW. Critically revised the manuscript: JG, FW, BW, VT, JR, HS, AK, DS, and OW. Gave the final approval to the version to be published: JG, JS, FW, BW, VT, JR, HS, AK, DS, and OW. All authors have read and approved the final version of the manuscript. Each of the authors acknowledges that he or she participated sufficiently in the work to take public responsibility for its content, and each of the authors agreed to be accountable for all aspects of the work in ensuring that questions related to the accuracy or integrity of any part of the work are appropriately investigated and resolved.

## Funding

This work was partially funded by the Free State of Thuringia and the European Social Fund (2016 FGR 0045) and the Deutsche Forschungsgemeinschaft (SFB1278 Polytarget). JG received a Carl-Zeiss-Stipend.

## Conflict of Interest Statement

The authors declare that the research was conducted in the absence of any commercial or financial relationships that could be construed as a potential conflict of interest.

## Abbreviations

AA, arachidonic acid; COX, cyclooxygenase; cPLA_2_, cytosolic phospholipase A_2_; DHA, docosahexaenoic acid; EPA, eicosapentaenoic acid; FLAP, 5-lipoxygenase-activating protein; LPS, lipopolysaccharide; GA, ginkgolic acid; HDHA, hydroxy-DHA; LM, lipid mediator; LO, lipoxygenase; LT, leukotriene; LX, lipoxin; mPGES-1, microsomal prostaglandin E_2_ synthase-1; NSAID, nonsteroidal anti-inflammatory drug; PD, protectin; PG, prostaglandin; Rv, resolvin; SPM, specialized pro-resolving mediator; TX, thromboxane.
