# Supplementary material for: Ginkgolic Acid is a Multi-Target Inhibitor of Key Enzymes in Pro-Inflammatory Lipid Mediator Biosynthesis
Source: Front Pharmacol. 2019 Jul 17;10:797. doi: 10.3389/fphar.2019.00797 (PMC6650749; doi:10.3389/fphar.2019.00797)
Supplement: Supplementary file 1 [file DataSheet_1.docx]

**Supporting Information**

**Ginkgolic acid is a multi-target inhibitor of key enzymes in pro-inflammatory lipid mediator biosynthesis**

**Jana Gerstmeier^1^, Julia Seegers^2^, Finja Witt^1^, Birgit Waltenberger^3^, Veronika Temml^3^, Judith M. Rollinger^4^, Hermann Stuppner^3^, Andreas Koeberle^1^, Daniela Schuster^5^, Oliver Werz^1^**

^1^Chair of Pharmaceutical/Medicinal Chemistry, Institute of Pharmacy, Friedrich-Schiller-University Jena, 07743 Jena, Germany;

^2^Department of Pharmaceutical Analytics, Pharmaceutical Institute, Eberhard-Karls-University Tuebingen, Auf der Morgenstelle 8, 72076 Tuebingen, Germany;

^3^Institute of Pharmacy / Pharmacognosy and Center for Molecular Biosciences Innsbruck (CMBI), University of Innsbruck, Innrain 80-82, 6020 Innsbruck, Austria;

^4^Department of Pharmacognosy, Faculty of Life Sciences, University of Vienna, Althanstraße 14, 1090 Vienna, Austria;

^5^Institute of Pharmacy, Department of Pharmaceutical and Medicinal Chemistry, Paracelsus Medical University Salzburg, Strubergasse 21, 5020 Salzburg, Austria.

**List of contents:**

S1. Results of the virtual screening of the CHM database with pharmacophore models M1 and M2

**S1.** **Results of the virtual screening of the CHM database with pharmacophore models M1 and M2**

Virtual screening of the CHM database comprising 10,216 compounds from medicinal preparations used in the traditional Chinese medicine (TCM) (Fakhrudin et al., 2010) with pharmacophore models M1 and M2 for mPGES-1 inhibitors (Waltenberger et al., 2011) achieved a hit rate of 0.04% (4 molecules) and 0.6% (61 molecules), respectively.

One of the 4 virtual hits recognized by M1 is the depside perlatolic acid, a lichen compound, which we proved to be a potent mPGES-1 inhibitor with an IC_50_ of 0.4 µM (Bauer et al., 2012).

M2, the partial query model of M1, allowing to omit either the aromatic ring feature or one of the hydrophobic features of M1, recognized the 4 molecules that were also identified by M1 (no omitted feature) and 57 virtual hits with one omitted feature, respectively. Many of these virtual hits were identified as depsides from lichen species, i.e., baeomycesic acid, barbatinic acid, diffrataic acid, evernic acid, gyrophoric acid, ramalic acid, and squamatic acid. One of these compounds, evernic acid, was subjected to biological testing and was shown to be moderately active on mPGES-1 with a residual activity of 53.9 ± 2.7% at 10 µM (Bauer et al., 2012).

The depsides physodic acid and olivetoric acid were also identified by M2, but within another virtual screening approach, since they were not comprised in the CHM database. Both omitted the aromatic ring feature. Interestingly, pharmacological testing revealed that they are also potent mPGES-1 inhibitors with IC_50_ values of 0.43 and 1.15 µM (Bauer et al., 2012), respectively, suggesting that the aromatic ring feature might not be important for the mPGES-1 inhibitory activity.

Ginkgolic acid (GA) is another virtual hit obtained from the virtual screening of the CHM database with pharmacophore model M2. It omitted the same feature as the highly active lichen constituents physodic acid and olivetoric acid. However, it comprises a different structural scaffold and was thus selected for pharmacological investigations within this study.

**References**

Bauer, J., Waltenberger, B., Noha, S.M., Schuster, D., Rollinger, J.M., Boustie, J., Chollet, M., Stuppner, H., and Werz, O. (2012). Discovery of depsides and depsidones from lichen as potent inhibitors of microsomal prostaglandin E2 synthase-1 using pharmacophore models. *ChemMedChem* 7**,** 2077-2081.

Fakhrudin, N., Ladurner, A., Atanasov, A.G., Heiss, E.H., Baumgartner, L., Markt, P., Schuster, D., Ellmerer, E.P., Wolber, G., Rollinger, J.M., Stuppner, H., and Dirsch, V.M. (2010). Computer-aided discovery, validation, and mechanistic characterization of novel neolignan activators of peroxisome proliferator-activated receptor gamma. *Mol Pharmacol* 77**,** 559-566.

Waltenberger, B., Wiechmann, K., Bauer, J., Markt, P., Noha, S.M., Wolber, G., Rollinger, J.M., Werz, O., Schuster, D., and Stuppner, H. (2011). Pharmacophore modeling and virtual screening for novel acidic inhibitors of microsomal prostaglandin E(2) synthase-1 (mPGES-1). *J Med Chem* 54**,** 3163-3174.
